# Risk of infective endocarditis and complicated infection in *Staphylococcus aureus* bacteremia – a retrospective cohort study on the role of bacteriuria

**DOI:** 10.1007/s10096-024-04850-7

**Published:** 2024-05-21

**Authors:** Oskar Bergenman, Bo Nilson, Magnus Rasmussen

**Affiliations:** 1 Kalmar Regional Hospital, Kalmar, Sweden; 2 Division of Infection Medicine, Department of Clinical Sciences, Lund University, BMC B14 SE-221 84, Lund, Sweden; 3 Clinical Microbiology, Office for Medical Services, Infection Prevention and Control, Region Skåne, Lund, Sweden; 4 Division of Medical Microbiology, Department of Laboratory Medicine Lund, Lund University, Lund, Sweden; 5 Department of Infectious Diseases, Skåne University Hospital, Lund, Sweden

**Keywords:** *Staphylococcus aureus*, *Staphylococcus aureus* bacteremia, *Staphylococcus aureus* Bacteriuria, Complicated *Staphylococcus aureus* bacteremia, Infective endocarditis

## Abstract

**Purpose:**

*S. aureus* bacteremia (SAB) is a common and severe infection with high mortality and morbidity. The clinical relevance of the finding of concurrent *S. aureus* bacteriuria (SABU) is debated. The goal of this study was to analyze whether a concurrent SABU is associated with complicated SAB, infective endocarditis (IE) and mortality.

**Methods:**

We conducted a retrospective cohort study, reviewing medical charts of all episodes of SAB in patients > 18 years in the region of Skåne, Sweden, between 1st of January and 31st of June 2020. Episodes where a concurrent urine culture was performed were included for analysis. An episode was considered as complicated SAB if there was either attributable mortality, recurrent infection, embolic stroke, or occurrence of a complicated focus of infection.

**Results:**

During the study period, there were 279 episodes of SAB. 154 episodes met the eligibility criteria, of whom 37 (24%) had concurrent SABU. In 78 episodes (51%), the patients had a complicated SAB. There was a significantly lower proportion of complicated SAB for episodes with concurrent SABU (32%), compared to episodes without concurrent SABU (56%), p-value 0.014. Moreover, in the cohort there were 11 episodes (7.1%) of IE and a 30 days mortality rate of 16%, with no difference between the groups with or without SABU.

**Conclusions:**

There is an association between concurrent SABU and a decreased risk for complicated SAB among patients with SAB. This study found no significant association between SABU and neither IE nor mortality for patients with SAB.

**Supplementary Information:**

The online version contains supplementary material available at 10.1007/s10096-024-04850-7.

## Introduction

*Staphylococcus aureus* is one of the most common causes of bacteremia and the most common cause of severe bloodstream infections, resulting in high morbidity and mortality worldwide [1–3]. In high income countries, the incidence of *S. aureus* bacteremia (SAB) ranges from 16 to 41/100 000 [4, 5], and despite intense research and advances in the treatment, the mortality remains at a high level (15–25%) [4].

SAB can present in several ways with different focal infections and symptoms ranging from mild fever alone to septic shock. The most common foci in SAB are skin- and soft tissue infections together with catheter related infection, however in 10–40% of cases there is no identified focus of infection [4, 6]. SAB is often referred to as being either complicated or uncomplicated. One commonly used definition of complicated SAB introduced by Fowler et al. [7] relies on either attributable mortality, recurrent infection, embolic stroke, or the occurrence of a complicated focus of infection. Risk factors for the SAB being complicated are community acquisition, skin manifestations of acute systemic infection, positive follow-up blood culture (BC) and persistent fever [7].

One of the most severe forms of complicated SAB is infective endocarditis (IE), and *S. aureus* is the leading cause of IE [4, 8, 9]. Among patients with SAB, 8–25% have IE [8, 10]. Various studies have investigated risk factors for developing IE for patients with SAB, resulting in scoring systems like VIRSTA, PREDICT and POSTIVE [10–12]. Another potential risk factor for severe infection, including IE, which none of the scoring systems have analyzed, might be concurrent *S. aureus* bacteriuria (SABU). Several studies have demonstrated an association with worse outcome in patients with SABU compared to patients without SABU. Examples of worse outcomes used by others include complicated SAB [13–15], admission to an intensive care unit (ICU) [13, 16], and mortality [14, 16–18]. One study has shown an association between concurrent SABU and IE, but only when excluding the cases of *S. aureus* caused urinary tract infection (UTI) from the SABU group [13]. Most studies have not found an association between SABU and IE [14–16, 19, 20].

*S. aureus* causes 1.3–3.2% of UTI with a positive urine culture (UC) [21–23], but in patients with indwelling catheter, *S. aureus* causes up to 12% of UTIs [24]. Some studies suggest that SAB develops secondary to SABU [25–28]. Nonetheless, it is generally believed that hematogenous seeding of bacteria to the kidneys followed by SABU is the most common reason for concomitant SAB and SABU. This notion is based on studies, where SAB has been linked to the development of pyelonephritis, renal abscess formation and *S. aureus* translocation to the urine [29–31].

For patients with SAB, the prevalence of SABU ranges from 7 to 39% in different studies [25, 32, 33]. The main predisposing factors for SABU are urinary catheterization, urinary tract obstruction and invasive procedures [25]. However, there are no guidelines on the clinical management of SABU in patients with SAB [25] and clinical experience indicates a lack of information on how to interpret such a finding. The aim of the current study is to analyze whether concurrent SABU is associated with complications in SAB including IE and mortality.

## Materials and methods

### Microbiology and data collection

We conducted a retrospective cohort study based on information from microbiology database and medical charts. All patients aged 18 years or above with at least one positive BC for *S. aureus* in the region of Skåne from the 1st of January to the 31st of June 2020 were eligible for the study. All microbiological samples in the region of Skåne, situated in the southernmost part of Sweden, are analyzed in the university hospital laboratory of Clinical Microbiology, Region Skåne, located in Lund. Thus, data from all ten hospitals in the region with a population of 1 388 910 (as of 2020) were collected for the study [34]. When a patient had multiple sets of BCs drawn, the BCs were grouped into episodes. An episode of SAB was initiated by the isolation of *S. aureus* from at least one BC bottle. An episode was considered ended if treatment was finished and there was a regress of symptoms. Any new positive BC after this was considered as a new episode. If a patient had more than one episode of SAB during the study period, all episodes of the patient were included. The patients were followed for 365 days through hospital medical records and population registries (for death) after the positive BC or until death occurred.

BCs were incubated in a BACTEC FX blood culture system (Becton Dickinson, Franklin Lakes, NJ, USA). Microflex LT/SH smart matrix-assisted desorption ionization-time of flight mass spectrometry (MALDI-TOF MS) (Bruker Daltonics, Bremen, Germany) was used for species identification, with FlexControl and MBT Compass 4.1 software (database MBT-BDAL-8468). A MALDI Biotyper score value of ≥ 2 was used as a cut-off for species determination [35].

For urine culturing, 10 µl of urine was smeared on a CNA agar plate with 4% horse blood (Neogen) and a Uriselect agar plate (Bio-Rad, Hercules, CA, USA), and then incubated at 35 °C in air over night. MALDI-TOF MS (Bruker Daltonics, Bremen, Germany) was used for species identification. 10^3^ colony forming units/milliliter was used as a cut-off value [36].

General data on patient age, gender, number of episodes and time to positivity (TTP) for the BC were collected from the laboratory information system of Clinical Microbiology, Region Skåne, Lund, Sweden. Then, medical charts were reviewed in patients where a urine culture had been performed within 48 h prior to 24 h post the BC that demonstrated SAB. Through medical records, information was collected regarding the blood and urine cultures, risk factors for SAB and SABU, focus of infection, diagnosis of IE and complicated SAB, mortality, and some general characteristics all according to the definitions below. A detailed list of the collected variables is presented in supplementary Table 1.

### Definitions

All time definitions are given as relation to the time of the initial BC demonstrating SAB.

A follow-up BC was considered performed if it was drawn within 48–96 h after the beginning of the treatment for SAB. If the initial set of positive BCs were positive also for any other bacteria it was considered a polymicrobial bacteremia, except if only one bottle of the BCs had growth of low-virulence skin bacteria, such as coagulase-negative staphylococci, as this was considered a contamination.

TTP was defined as the time from placement of a vial in the incubator to the signal of positivity. The shortest TTP of cultures drawn the day as the start of the SAB was used. For polymicrobial cultures, TTP was not recorded [10]. Any UC positive for *S. aureus* within 48 h prior to 24 h after the BC demonstrating SAB, was regarded as the patient having concurrent SABU. UCs with mixed flora not specifying if *S. aureus* was identified were not regarded as SABU.

Earlier urinary tract surgery was defined as any surgery of the urinary tract within 7 days prior to the BC. Urinary tract obstructive pathology was defined as either a medical history of treatment for obstruction of the urinary tract in the last year, or presence of an underlying obstructive disease such as urethral stones or prostate hyperplasia. Urinary tract catheterization included any use of urethral catheterization within the last 7 days, regardless of the duration (including clean intermittent catheterization). Symptoms of a UTI were defined according to Centers for Disease Control and Prevention [37] as the presence of any of the symptoms: suprapubic tenderness, costovertebral pain, increased urinary frequency, urgency or dysuria. Fever was excluded from the definition since all the patients had a concurrent SAB. Pyuria and inflamed area around a nephrostomy were considered signs of UTI. Information about UTI-symptoms were collected from 48 h prior to 24 h after the positive BC. A patient was considered to have a focal infection, including UTI, if meeting at least two of the following three criteria (at the same location) during the SAB episode; (1) Imaging result compatible with a focal infection. (2) Signs or symptoms of a focal infection. (3) A culture positive for *S. aureus* from the location.

Comorbidities were classified using the updated Charlson comorbidity score [38]. The infection was considered nosocomial if the positive BC was gathered after more than 48 h of hospitalization. Health care-associated infection was defined according to Friedman et al. [39].

Sepsis was defined according to the Sepsis-3 criteria [40]. An underlying organ failure prior to the infection excluded that organ from receiving any sequential organ failure assessment (SOFA)-score. The highest documented value within 6 h of the positive BC was used to calculate the SOFA-score.

### Outcome

A complicated SAB was defined according to the definition by Fowler et al. [7] as the presence of either; (1) Attributable mortality, (2) A complicated focal infection during the hospitalization, (3) Embolic stroke, or (4) Recurrent infection within 12 weeks after the episode. Attributable mortality was defined as patients dying with persistent signs, symptoms or treatment for the SAB. Complicated infections included IE, septic arthritis, vertebral osteomyelitis, epidural abscess, deep tissue abscess, septic thrombophlebitis, meningitis, pneumonia, and empyema [7]. These diagnoses were established using the definition for focal infections except for IE where each episode was defined as being either definitive, possible, or rejected IE according to the Duke-ISCVID criteria [41]. Only definitive IE was considered as IE.

30 days mortality was defined from the time of the positive BC demonstrating *S. aureus* until death occurred.

### Statistical analyses

All statistical analyzes were performed using Statistical Package for Social Sciences, version 28 for Macintosh (IBM Corp. Armonk, New York. Released 2021). Continuous variables were presented as median and interquartile range. Categorical variables were presented as the number of cases and % of total. Mann Whitney U-test was used to compare difference between SABU and non-SABU for continuous variables, including age, TTP, days of antibiotics and SOFA-score, since the data was not considered to be normally distributed. Shapiro-Wilk test was performed to investigate normal distribution. Fischer’s exact test or Person’s chi-square was performed to compare categorical variables. A p-value of < 0.05 was considered statistically significant.

## Results

**Fig. 1:**
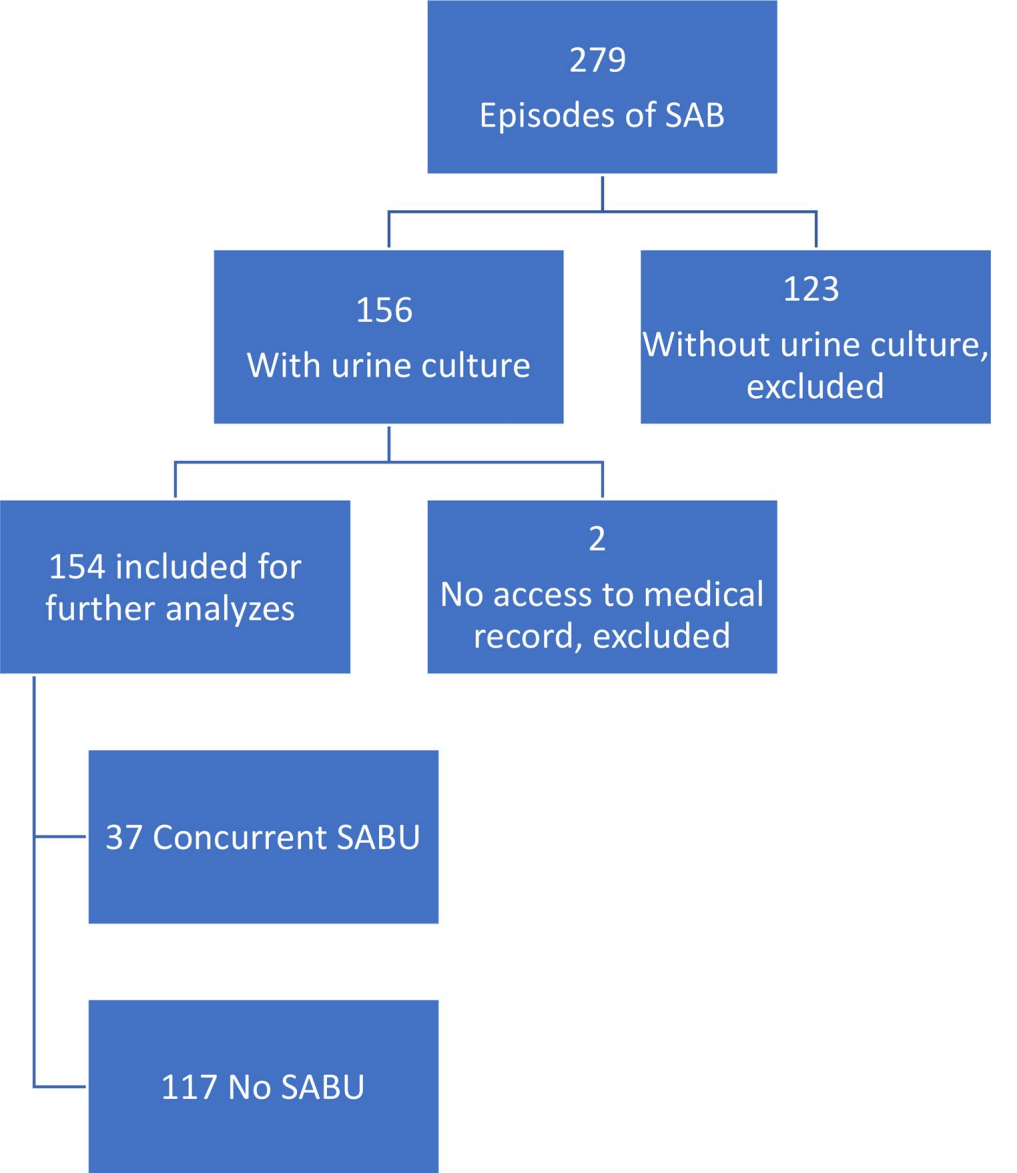
A flow chart showing the included and excluded episodes of SAB in the study. SAB, *Staphylococcus aureus* bacteremia; SABU, *Staphylococcus aureus* bacteriuria

During the study period, there were 279 unique episodes of SAB in patients above 18 years of age. In 156 of episodes, there was a concurrent UC drawn. Two episodes were excluded since there were no medical records available. Out of 154 episodes, in 153 patients, 37 episodes (24%) had concurrent SABU whereas in 117 episodes the UC was negative. A flowchart of the inclusions and exclusions is presented in Fig. 1.

Patient characteristics for the 154 episodes are presented in Table 1, both as a total and as the two subgroups SABU and non-SABU. The median age was 74 years (66–83), and in 98 episodes (64%) the patients were male. Patients had a higher Charlson comorbidity score in the SABU group, compared to non-SABU (p-value 0.040). Indwelling catheter was more common among patients with concurrent SABU, as was urinary tract obstruction. TTP, was lower in the SABU group, median 12 (14–16), than in the non-SABU group, median 14 (10–17), p-value 0.012 (Table 1).

**Table 1:** Patient characteristics for episodes with and without bacteriuria

Variable	All SAB	SABU	Non-SABU	*P*-value
	*n* = 154	*n* = 37	*n* = 117	
Age, years*	74 (66–83)	74 (66–84)	74 (66–83)	0.80
Male sex	98 (64%)	28 (76%)	71 (61%)	0.12
Charlson Comorbidity Score				**0.040**
0–1	68 (44%)	13 (35%)	55 (47%)	
2–3	47 (31%)	9 (24%)	38 (33%)	
≥4	38 (25%)	15 (41%)	23 (20%)	
Mode of acquisition				0.87
Community	46 (30%)	11 (30%)	35 (30%)	
Health-care-associated	78 (51%)	20 (54%)	58 (50%)	
Nosocomial	29 (19%)	6 (16%)	23 (20%)	
Risk factors for SABU				
Catheter	47 (31%)	24 (65%)	23 (20%)	**< 0.001**
Surgery	2 (1.3%)	2 (5.4%)	0	0.057
Obstruction	32 (21%)	15 (41%)	17 (15%)	**0.002**
Persistent bacteremia	26 (17%)	3 (8.1%)	23 (20%)	0.15
TTP*	13 (10–16)	12 (10–14)	14 (10–17)	**0.012**
Known focus	93 (60%)	26 (70%)	67 (57%)	0.18

Unless otherwise noted, data is presented as numbers (%) of cases within the groups

*Abbreviations* used were SABU, *Staphylococcus aureus* bacteriuria; SAB *Staphylococcus aureus* bacteremia; TTP, time to positivity

*Data presented as median (interquartile range)

P-value originates from Chi^2^, Fischer’s exact test, or Man Whitney U-test (age, TTP) to compare SABU versus non-SABU. P-value of < 0.05 was considered statistically significant and such values are given in bold

### Outcome - complicated *S. aureus* bacteremia

In 78 episodes (51%), the patients had a complicated SAB. There was a significantly lower proportion of complicated cases for episodes with concurrent SABU (12 cases (32%)), compared to episodes without concurrent SABU (66 cases (56%)), p-value 0.014 with Fischer’s exact test. The results are depicted in Table 2. When exploring why the episodes were classified as complicated SAB, it was found that the dominating reason was a complicated focus of infection, both for episodes with concurrent SABU (8 cases (22%)) and without concurrent SABU (53 cases (45%), Table 2). A detailed description of the focus of the infections is given in Supplementary Table 2.

**Table 2:** Complicated SAB as outcome

Outcome	All SAB	SABU	Non-SABU	*P*-value
	*n* = 154	*n* = 37	*n* = 117	
Complicated SAB	78 (51%)	12 (32%)	66 (56%)	**0.014**
Attributable mortality	22 (14%)	4 (11%)	18 (15%)	0.60
Recurrent infection	4 (2.6%)	1 (2.7%)	3 (2.6%)	1.0
Stroke	4 (2.6%)	1 (2.7%)	3 (2.6%)	1.0
Complicated focus of infection	61 (40%)	8 (22%)	53 (45%)	**0.012**
IE	11 (7.1%)	4 (11%)	7 (6.0%)	0.30
Abscesses	25 (16%)	6 (16%)	19 (16%)	1.0
Bone and joint infections	13 (8.4%)	1 (2.7%)	12 (10%)	0.19
Respiratory tract infections	14 (9.1%)	0	14 (12%)	**0.023**
Thrombophlebitis	12 (7.8%)	0	12 (10%)	0.071

Data is presented as numbers (%) of episodes within the groups

*Abbreviations* used were SABU, *Staphylococcus aureus* bacteriuria; SAB *Staphylococcus aureus* bacteremia; IE, Infective endocarditis

P-value originates from Fischer’s exact test to compare SABU versus non-SABU. P-value of < 0.05 was considered statistically significant and are given in bold

### Outcome – infective endocarditis and mortality

In total, there were 11 episodes (7.1%) of IE in the study. The proportion did not differ significantly between the groups, with 4 cases (11%) in the SABU group, and 7 cases (6.0%) in the non-SABU group (Table 3). Native valve IE was most common.

**Table 3:** The outcome IE, divided into different subgroups of the disease

Outcome	All SAB	SABU	Non-SABU	*P*-value
	*n* = 154	*n* = 37	*n* = 117	
IE	11 (7.1%)	4 (11%)	7 (6.0%)	0.30
NVE	5 (3.2%)	3 (8.1%)	2 (1.7%)	0.09
RSIE	1 (0.6%)	0	1 (0.9%)	1.0
PVE	2 (1.3%)	1 (2.7%)	1 (0.9%)	0.42
CDRIE	2 (1.3%)	0	2 (1.7%)	1.0
Unclassified	1 (0.6%)	0	1 (0.9%)	1.0

Data is presented as numbers (%) of cases within the groups

*Abbreviations* used were IE, Infective endocarditis; NVE, Native valve endocarditis; RSIE, Right-sided infective endocarditis; PVE, Prosthetic valve endocarditis; CDRIE, Cardiac device related infective endocarditis

P-value originates from Fischer’s exact test to compare SABU versus non-SABU. P-value of < 0.05 was considered statistically significant

The 30 days mortality was 16% for the study population and did not differ either between the groups, 16% for SABU vs. 15% for non-SABU. A Kaplan-Mayer survival analysis was performed (supplementary Fig. 1) and did not demonstrate a significant difference between the groups.

## Discussion

In the region of Skåne, there were 37 episodes (24%) of concurrent SABU among patients with SAB where a UC had been taken during the study period. This is in range with the proportion of SABU found in several other studies [25, 33, 42]. Urethral catheterization and urinary tract obstruction were found to be risk factors for concurrent SABU, as previously described [19].

This study found an association between concurrent SABU and a decreased risk for complicated SAB. This is contrary to the increased risk for complicated SAB found by others [13–15]. However, none of these studies used Fowler’s [7] definition for complicated SAB. Pulcini et al. [15] used only complicated foci in the definition, and not the same ones - where osteomyelitis, endophthalmitis and prostatitis were added and a few deleted compared to Fowler. Perez-Jorge et al. [14] used a wider definition, including almost the same foci as Pulcini, but also septic shock, persistent SAB (lasting more than 5 days after starting adequate *S. aureus* treatment), and recurrent SAB. Asgeirsson et al. [13] likewise used persistent bacteremia, a criterium that Fowler considered a risk factor rather than an outcome. Asgeirsson et al. also included all secondary foci remote from the initial focus and furthermore all unknown foci. Moreover, they only found an association between complicated SAB and SABU when excluding the patients with UTI from the SABU group. Thus, a likely explanation to the association between SABU and decreased risk for complicated SAB in our study is the way that we chose to define complicated SAB. Importantly, in the SABU group of our study, more than half of the patients had UTI as a focus, and UTI was not considered a complicated infection. No patients in the SABU-group had pneumonia or septic thrombophlebitis, both defining a complicated SAB, which were the most common foci in the non-SABU group.

In this study, we could not find any association between concurrent SABU and mortality, something which has been reported by other studies [14, 16–18]. Some studies used 1-year mortality, some used only in-hospital mortality whereas the two studies that specifically compared 30 days mortality, like our study, failed to show an association to SABU [13, 19]. The current study has however, due to a relatively small sample size, a limited power to detect potential associations between SABU and mortality.

Furthermore, the current study could not find a significant difference in the proportion of episodes with IE between patients with SABU and non-SABU. The proportion of IE was however higher in the SABU group, and it is possible that a difference could have been detected if the study population was made much larger. In possible support of such a speculation was the finding that TTP was shorter in the SABU-group. A shorter TTP has previously been associated with IE in SAB [10]. However, the results on no association to IE in this study is consistent with several other studies which also did not find such associations [14–16, 19, 20, 42]. Only one retrospective study and one meta-analysis has shown an association between IE and SABU, and this could only be demonstrated when excluding patients with UTI from the analysis [13, 42]. We believe that both the hematogenous spread of *S. aureus* to the kidney and subsequently to the urine and ascending UTI causing pyelonephritis can lead to symptoms of UTI. Therefore, we chose not to exclude patients with symptoms of UTI from this analysis. Interestingly, if we would have excluded the patients with UTI, there would have been only one episode of IE in the SABU group instead of four.

A shortcoming of this study, as in all retrospective medical chart-studies, is the risk for errors in classification. Another limitation is that the study only included patients with SAB who also underwent a UC. This might result in a sample bias with altered rate in for example urinary surgery, where one could hypothesize that a physician is more likely to take a UC. Nonetheless, we have no way of knowing why these patients underwent a UC or not, and consequently our results are not generalizable for all patients with SAB. The results may also not be representative for other health care systems outside Skåne, where there are different routines and indications for when to perform a UC or BC. We are aware that antibiotic treatment can possibly have been initiated for the SAB could have impacted the results from UC, and false negative results could lead to detection of fewer episodes of SABU. The demand that the UC should be taken a maximum of 24 h post the BC was used to lower this potential impact.

A strength of the study is the consistency of the methods of BC and UC, since one single laboratory makes all the analyses for the entire region, as mentioned earlier. Since this is a population-based study, the study also has an increased generalizability compared to single center studies.

In conclusion, there is an association between concurrent SABU and a decreased risk for complicated SAB among patients with SAB who had had a UC drawn in our region. The reason for this might be because UTI, the most common focus in episodes with SAB and concurrent SABU, was not considered a complicated focus, whereas septic thrombophlebitis and pneumonia, the most common focus of episodes of SAB without SABU, were considered complicated. Moreover, this study found no significant association between SABU and either IE or 30 days mortality rate in patients with SAB.

### Electronic Supplementary Material

Below is the link to the electronic supplementary material.

Supplementary Material 1

## Data Availability

Pseudonyonymized data will be made available upon reasonable request.
