# Supplementary material for: Risk of infective endocarditis and complicated infection in Staphylococcus aureus bacteremia – a retrospective cohort study on the role of bacteriuria
Source: Eur J Clin Microbiol Infect Dis. 2024 May 21;43(7):1419–26. doi: 10.1007/s10096-024-04850-7 (PMC11271437; doi:10.1007/s10096-024-04850-7)
Supplement: Supplementary file 1 — Supplementary Material 1 [file 10096_2024_4850_MOESM1_ESM.docx]

**Supplementary Table 1.** A detailed list of all the variables collected for each episode of SAB

| Category | Variables collected |
| --- | --- |
| General | Patient’s age; Patient’s gender; Community-acquired SAB; Health care-associated SAB; Nosocomial SAB |
| Mortality | Deceased within 30 days?; Numbers of days until decease; In hospital-mortality |
| Charlson Comorbidity Score | Congestive heart failure; Dementia; Chronic pulmonary disease; Rheumatologic disease; Mild liver disease; Moderate/severe liver disease; Diabetes with complications; Moderate/severe renal disease; Hemiplegia or paraplegia; Any malignancy; Metastatic solid tumor; AIDS |
| Sepsis (SOFA score) | Respiration-score; Coagulation-score; Liver-score; Cardiovascular score; Central nervous system score; Renal score |
| SAB, including risk factors | TTP for BC demonstrating SAB; Follow-up BC collected within 48–96 hours?: Follow-up BC positive for SA?; Previous SAB within 2 years; Hemodialysis; Permanent iv catheter; Port-a-cath |
| SABU, including risk factors | UC positive for SA?; Amount of growth of SA; Earlier surgery in urinary tract; Urinary tract catheterization; Urinary tract obstruction disease |
| UTI | Suprapubic tenderness; Costovertebral pain; Increased urinary frequency; Urgency; Dysuria; Sign of UTI (pyuria, inflamed nephrostomy) |
| Focus of infection | Positive imaging indicating a focus; Focal signs or symptoms; SA found in focal culture |
| Complicated SAB | Endocarditis according to Duke-ISCVID criteria; Septic arthritis; Deep tissue abscess; Vertebral osteomyelitis; Epidural abscess; Septic thrombophlebitis; Meningitis; Other focus considered complicated; Attributable mortality; Recurrent infection; Embolic stroke |


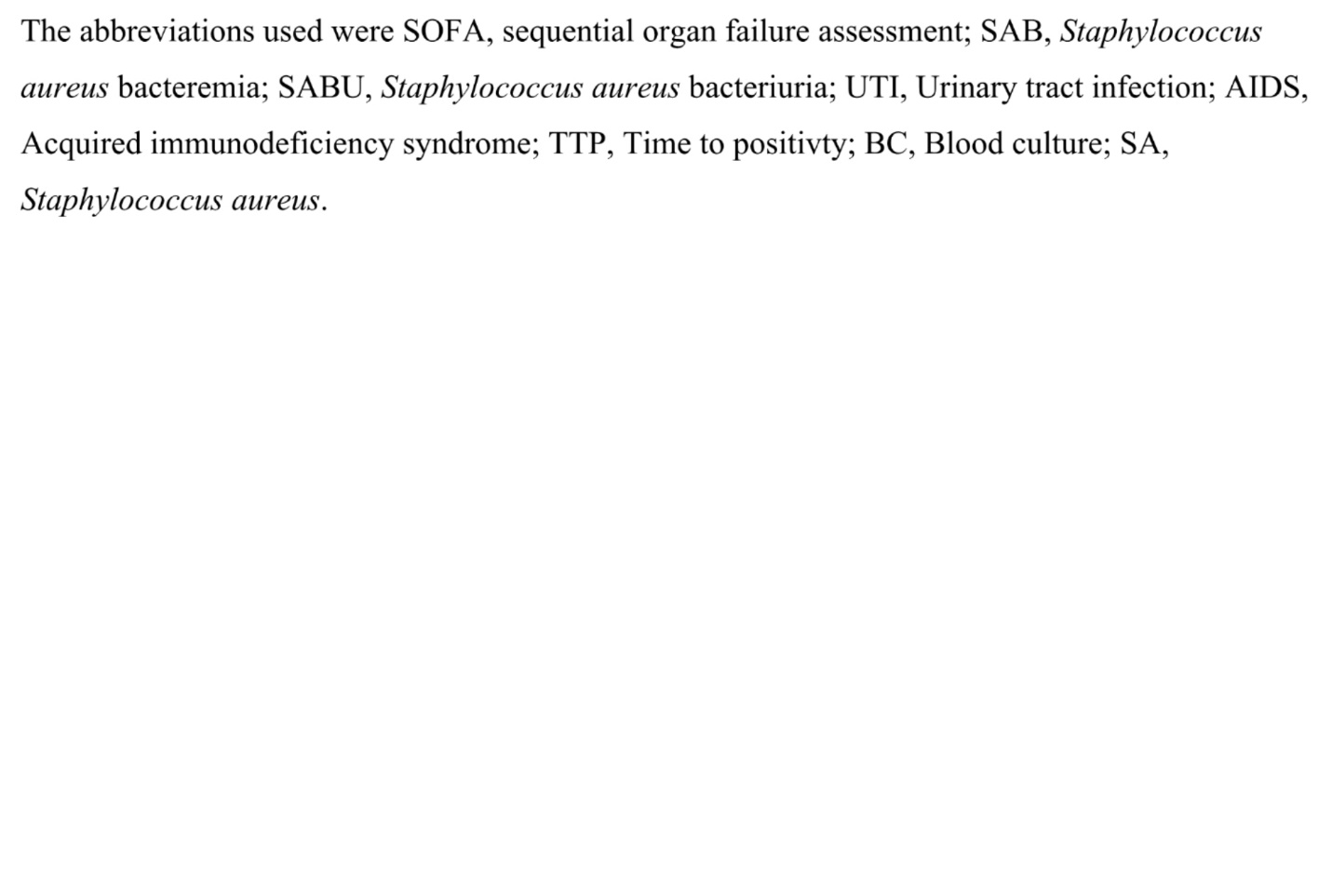


**Supplementary Table 2.** A description of the foci of infection

| Focus | SABU  n = 37 | Non-SABU  n = 117 |
| --- | --- | --- |
| Complicated ones |  |  |
| IE | 4 (11%) | 7 (6.0%) |
| Septic arthritis | 1 (2.7%) | 5 (4.3%) |
| Vertebral osteomyelitis | 0 | 7 (6.0%) |
| Epidural abscess | 4 (11%) | 6 (5.1%) |
| Deep tissue abscess | 2 (5.4%) | 13 (11%) |
| Septic thrombophlebitis | 0 | 12 (10%) |
| Pneumonia | 0 | 12 (10%) |
| Empyema | 0 | 2 (1.7%) |
| Uncomplicated ones |  |  |
| Skin and wound infections | 3 (8.1%) | 10 (8.5%) |
| Osteitis | 0 | 3 (2.6%) |
| UTI | 20 (54%) | 0 |
| Others* | 0 | 6 (5.1%) |
| Not known focus | 11 (30%) | 50 (43%) |

In 2 episodes in the SABU group and in 1 episode in the non-SABU group there were more than 1 foci, hence the sum of the foci is greater than the number of episodes in the group.

* Others include colitis, cholecystitis, parotitis, and infection in the dose pocket of pacemaker and port-a-cath. The abbreviations used were SABU, *Staphylococcus aureus* bacteriuria; UTI, urinary tract infection.


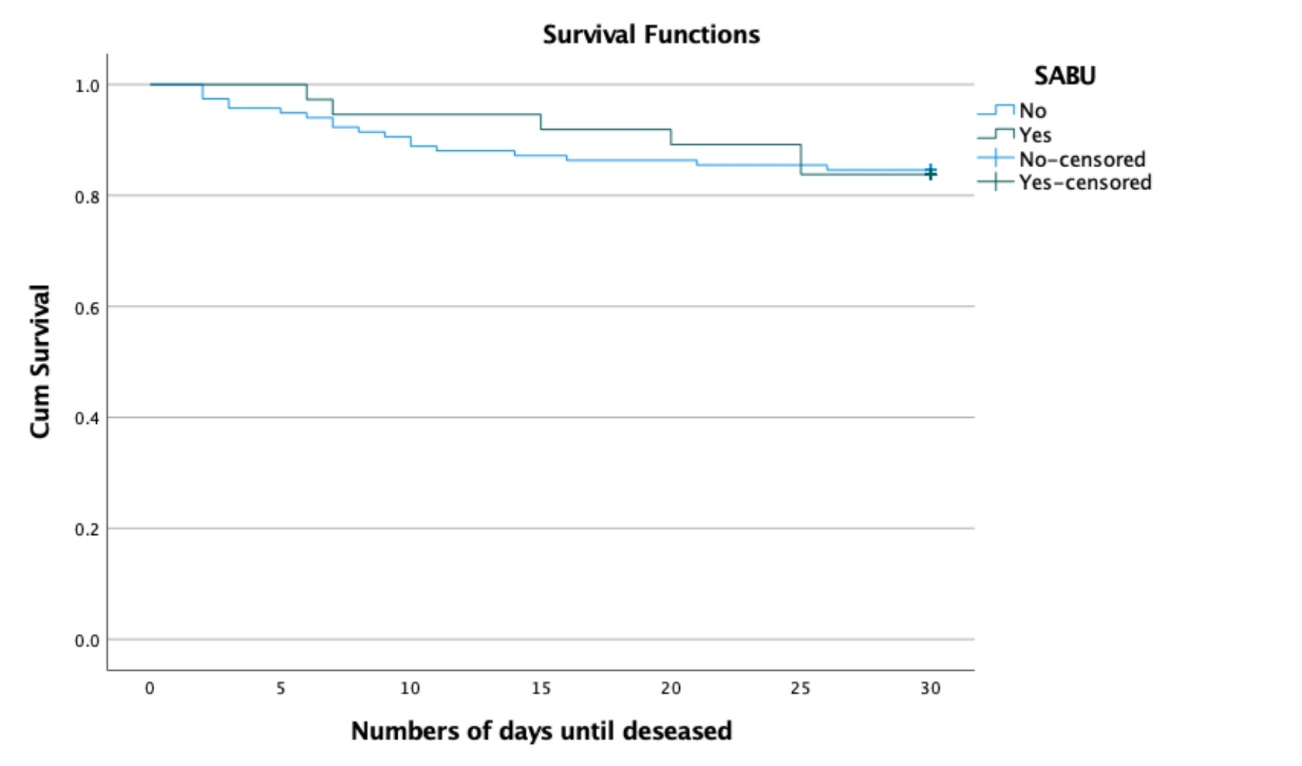


**Supplementary Fig. 1.** A graph showing a Kaplan-Meier survival analysis for the 30 days mortality rate, comparing patients with and without bacteriuria. The difference between the groups was non-significant, where a P-value of < 0.05 was considered statistically significant. SABU, *Staphylococcus aureus* bacteriuria.
